# SCO-spondin, a giant matricellular protein that regulates cerebrospinal fluid activity

**DOI:** 10.1186/s12987-021-00277-w

**Published:** 2021-10-02

**Authors:** Vania Sepúlveda, Felipe Maurelia, Maryori González, Jaime Aguayo, Teresa Caprile

**Affiliations:** Departamento de Biología Celular, Facultad de Ciencias Biológicas, Universidad de Concepción, Concepción, Chile

**Keywords:** Cerebrospinal fluid, LDL receptor family, Matricellular protein, Reissner fiber, SCO-spondin, Subcommissural organ, Thrombospondin repeats

## Abstract

Cerebrospinal fluid is a clear fluid that occupies the ventricular and subarachnoid spaces within and around the brain and spinal cord. Cerebrospinal fluid is a dynamic signaling milieu that transports nutrients, waste materials and neuroactive substances that are crucial for the development, homeostasis and functionality of the central nervous system. The mechanisms that enable cerebrospinal fluid to simultaneously exert these homeostatic/dynamic functions are not fully understood. SCO-spondin is a large glycoprotein secreted since the early stages of development into the cerebrospinal fluid. Its domain architecture resembles a combination of a matricellular protein and the ligand-binding region of LDL receptor family. The matricellular proteins are a group of extracellular proteins with the capacity to interact with different molecules, such as growth factors, cytokines and cellular receptors; enabling the integration of information to modulate various physiological and pathological processes. In the same way, the LDL receptor family interacts with many ligands, including β-amyloid peptide and different growth factors. The domains similarity suggests that SCO-spondin is a matricellular protein enabled to bind, modulate, and transport different cerebrospinal fluid molecules. SCO-spondin can be found soluble or polymerized into a dynamic threadlike structure called the Reissner fiber, which extends from the diencephalon to the caudal tip of the spinal cord. Reissner fiber continuously moves caudally as new SCO-spondin molecules are added at the cephalic end and are disaggregated at the caudal end. This movement, like a conveyor belt, allows the transport of the bound molecules, thereby increasing their lifespan and action radius. The binding of SCO-spondin to some relevant molecules has already been reported; however, in this review we suggest more than 30 possible binding partners, including peptide β-amyloid and several growth factors. This new perspective characterizes SCO-spondin as a regulator of cerebrospinal fluid activity, explaining its high evolutionary conservation, its apparent multifunctionality, and the lethality or severe malformations, such as hydrocephalus and curved body axis, of knockout embryos. Understanding the regulation and identifying binding partners of SCO-spondin are crucial for better comprehension of cerebrospinal fluid physiology.

## Introduction

Cerebrospinal fluid (CSF) is a clear fluid that occupies the ventricular and subarachnoid spaces inside and around the brain and spinal cord. CSF plays an essential role in the homeostasis of the central nervous system (CNS) and its composition must be finely tuned to establish a stable internal milieu. It provides buoyancy and protection to the brain and spinal cord; it transports nutrients, neuroactive substances, and even waste substances for clearance over the entire CNS; and it regulates brain volume, neurogenesis, behavior, and sleep/wake cycles [1–5].

The appearance of the CSF is concomitant with neural tube formation, a shared feature in all vertebrates. At this early stage, amniotic fluid gets trapped inside the neural tube and constitutes the earliest embryonic CSF (eCSF) [6]. After the appearance of this sealed cavity inside the primordial CNS, the composition of the eCSF changes as the embryo matures, adapting to the CNS requirements. eCSF impacts neuroepithelial cells by the trophic influence of various factors that regulate their survival, proliferation, and differentiation [4, 7, 8] (Table 1).

**Table 1:** Relevant CSF molecules and their ascribed functions

CSF component	Function	References
Amyloid-β peptide	Neurodegeneration	[190]
BMPs	Differentiation, proliferation, and survival of neuroepithelium	[6, 191–193]
Clusterin (ApoJ)	Neurodegeneration	[194]
Epithelial growth factor	Proliferation and differentiation of neuroepithelium	[78]
Fibroblast growth factor 2	Proliferation, differentiation, and survival of neuroepithelium	[78, 195]
Insulin growth factor 1	Differentiation, proliferation, and survival of neuroepithelium. Adult neurogenesis, synaptogenesis	[191, 196–198]
Lipoproteins (LDL, VLDL, HDL)	Proliferation and differentiation of neuroepithelium	[106, 199]
Monoamines (Epinephrine, norepinephrine, serotonin, L-Dopa)	Neurotransmission	[200]
Nerve growth factor	Cell proliferation and survival of neuroepithelium	[201]
Reelin	Brain development	[202]
Retinoic acid	Differentiation of neuroepithelium	[203]
Shh	Differentiation, proliferation, and survival of neuroepithelium	[204]
SCO-spondin	Differentiation, proliferation, and survival of neuroepithelium	[24, 25, 205]
Transforming growth factor β1–β2	Regeneration and pathological processes	[206, 207]
Wnts (Wnt4, 5A)	Differentiation and proliferation of neuroepithelium, brain morphogenesis	[109, 191, 208, 209]

The molecules listed were chosen because they bind to proteins with similar domains as those present in SCO-spondin

The neurogenic and proliferative activities of some of these factors have been reported by inhibition in vivo and in vitro (Table 2). These studies showed that although the eCSF contains numerous factors, the inhibition of any of them dramatically affects neuroepithelium development, suggesting that eCSF is not merely a sum of molecules with independent effects. In contrast, these molecules must be acting in a coordinated and interrelated manner. The same interrelation occurs within the classical extracellular matrix (ECM) that facilitates the interaction between different molecules and serves as a reservoir and regulator of different morphogens via the action of matricellular proteins.

**Table 2:** Effect of inhibition of individual eCSF factors on the differentiation and proliferation of neuroepithelium

CSF component	Inhibition approach	Inhibition versus control	References
Lipoproteins	Chick mesencephalic explants cultured in eCSF depleted of lipoproteins	80% lower neurodifferentiation 90% lower proliferation	[106]
FGF2	Addition of anti-FGF2 to chick mesencephalic explants cultured in eCSF	40% lower neurodifferentiation 60% lower proliferation	[195]
SCO-spondin	Addition of anti-SCO-spondin to chick mesencephalic explants cultured in eCSF	75% lower neurodifferentiation 275% higher proliferation	[24, 81]
Retinoic acid/retinol binding protein (RBP)	Addition of anti-RBP to chick mesencephalic explants cultured in eCSF	40% lower neurodifferentiation	[203]

The summation of individual effects is higher than 100%, revealing that CSF factors have interactive effects

**Fig. 1:**
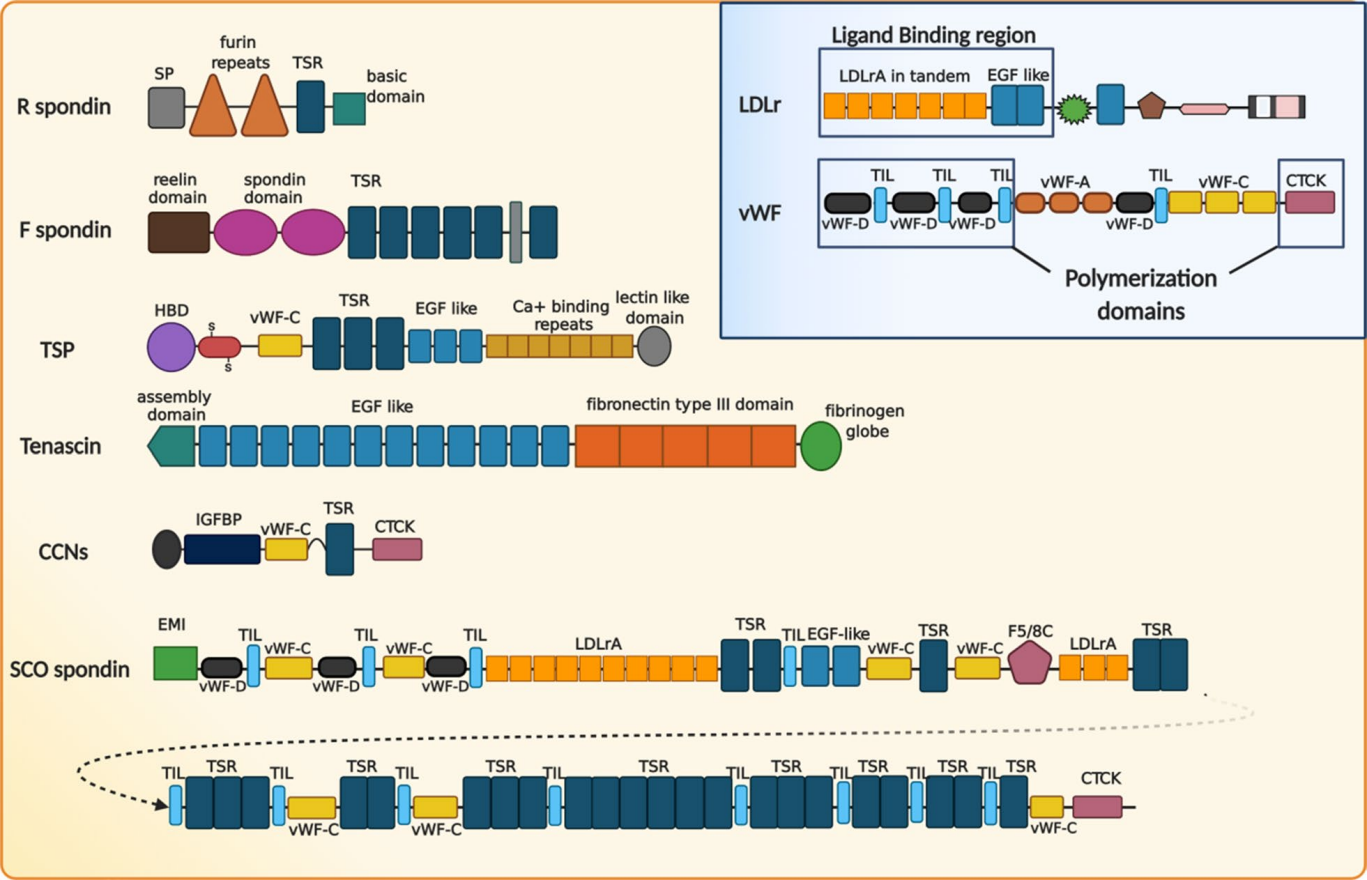
Schematic representation of matricellular proteins, LDL receptor, and von Willebrand factor compared with SCO-spondin. Yellow box: Matricellular proteins are modular proteins, with a high prevalence of TSR, vWF-C, EGF-like, and CTCK domains. The SCO-spondin structure shares homology with several matricellular proteins, especially TSP and CCNs. Blue box: Domain structure of the LDL receptor and vWF. Both proteins have structural similarities with SCO-spondin, particularly in the ligand-binding region of the LDL receptor family (several LDLrA in tandem and 2 EGF-like domains) and in the domains responsible for polymerization of vWF (3 vWF-D domains followed by TIL domains at the N-terminus and a CTCK domain at the C-terminus). *CTCK* Carboxyl-terminal cystine knot, *EGF* Epidermal growth factor, *EMI* Elastin microfibril interface domain, *HBD* Heparin-binding domain, *IGFBP* insulin-like growth factor-binding protein, *LDLr* Low density lipoprotein receptor, *SP* Signal peptide, *TSR* Thrombospondin repeat, *TSP* Thrombospondin, *vWF* von Willebrand factor, *vWF-A,C and D* von Willebrand factor domain type A,C and D

Matricellular proteins are modular extracellular proteins with the ability to interact with different ligands, including growth factors, cytokines, proteases, and cell receptors [9]. These proteins act as integrators or modulators of extracellular signals, and their function is variable depending on the combination of available cell-surface and extracellular ligands [10]. The group of matricellular proteins includes thrombospondin (TSP) 1–5, tenascins (TNC), R-spondin, F-spondin, and CCN family (for Connective tissue growth factor (CTGF), Cysteine rich protein (Cyr61), and Nephroblastoma overexpressed gene (Nov)), among others [11] (Fig. 1).

**Fig. 2:**
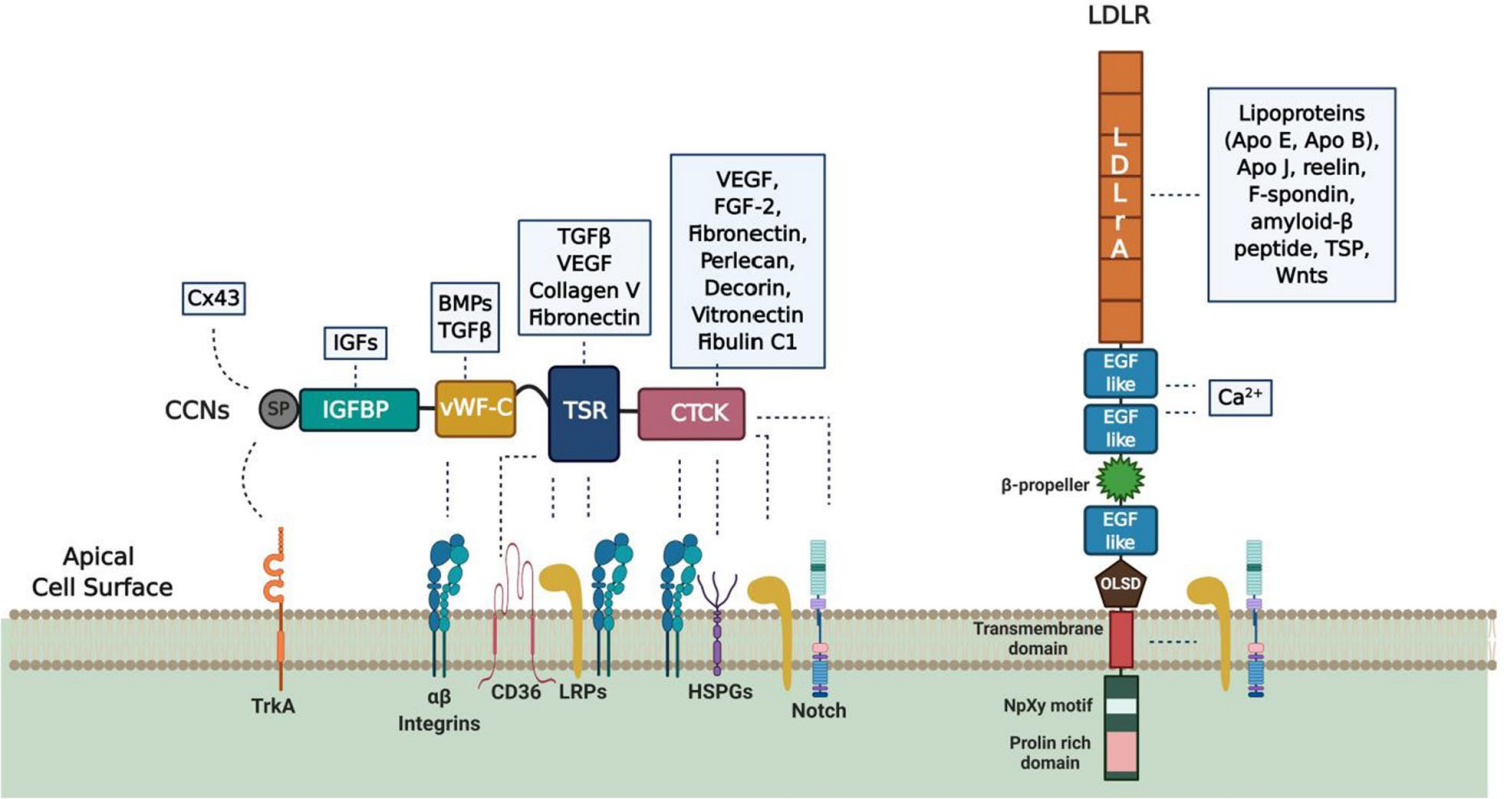
Schematic diagram of the domain structures of CCN-1 and LDL receptor showing their canonical interactions. Left) CCN-1 consist of insulin-like growth factor-binding protein (IGFBP), von Willebrand factor type C repeat (vWF-C), thrombospondin type I repeat (TSP), and a carboxyl-terminal cystine knot motif (CTCK) domain. The locations of identified interaction with cell receptors and soluble factors are shown in the diagram (modified from [228]). Right) The ligand-binding region of the LDL receptor is formed by several LDLrA domains in tandem, followed by 2 EGF-like domains. This region, also present in SCO-spondin, binds several molecules shown in the image and is characteristic and conserved in all members of the LDLr family (Modified from [103]). *BMP* Bone morphogenetic protein, *CTCK* Carboxyl-terminal cystine knot, *EGF* Epidermal growth factor, *EMI* Elastin microfibril interface domain, *HBD* Heparin-binding domain, *HSPG* Heparan sulfate proteoglycan, *IGF* insulin-like growth factor, *IGFBP* insulin-like growth factor-binding protein, *LDLr* Low density lipoprotein receptor, *LRP* LDLr-related protein, *TGFβ* Transforming growth factor β, *TSR* Thrombospondin repeat, *TrkA* Tyrosine kinase A, *TSP* Thrombospondin, *VEGF* Vascular endothelial growth factor, *vWF* von Willebrand factor, *vWF-A,C and D* von Willebrand factor domain type A,C and D

The size of matricellular proteins is diverse, with tenascin being the largest, with a monomeric size of ~ 250 kDa and oligomers of over a million Daltons, whereas CCN-1 only 35–40 kDa. Independently from their size, a common characteristic of matricellular proteins is their modular structure. Some domains are shared among several matricellular proteins, such as the epidermal growth factor (EGF)-like domain, von Willebrand factor type-C domain (vWF-C), thrombospondin type I repeat (TSR), and a carboxyl-terminal cystine knot (CTCK) motif (Fig. 1). Each of these domains has the potential to bind to extracellular proteins and cell-surface receptors [11, 12] (Fig. 2). For instance, CCN proteins interact with several cell receptors of the integrin family, low density lipoprotein receptor (LDLr) related proteins, contactin, or heparan sulfate proteoglycan (HSPG), as well as with soluble factors, such as bone morphogenetic proteins (BMPs), and family members or vascular endothelial growth factor (VEGF), fibroblastic growth factor (FGF) and transforming growth factor-β (TGFβ) (Fig. 2) [13, 14]. The diversity of the binding partners leads to a comparison of these proteins with a centralized coordination network [15]. The role of matricellular proteins in the CNS is an area of intense research, revealing that they are mainly involved in processes that require remodeling events, such as development, synaptogenesis, injury and in CNS disorders [16–20].

To date, the occurrence of matricellular proteins in CSF that account for the interrelationship between the different components of CSF has not been described. However, the existence of some type of “sensor” that controls eCSF homeostasis was suggested by Parvas et al. [21, 22], whom analyzed the eCSF concentration of FGF-2 and retinol binding protein (RBP) before and after the injection of these compounds into the eCSF, and found that surprisingly the concentration of these compounds did not increase, but remained stable after injection. These results may be explained by the occurrence of a CSF mechanism, such as matricellular proteins, with the capacity to trap different molecules when concentration exceeds homeostatic levels and release them when the concentration diminishes.

**Fig. 3:**
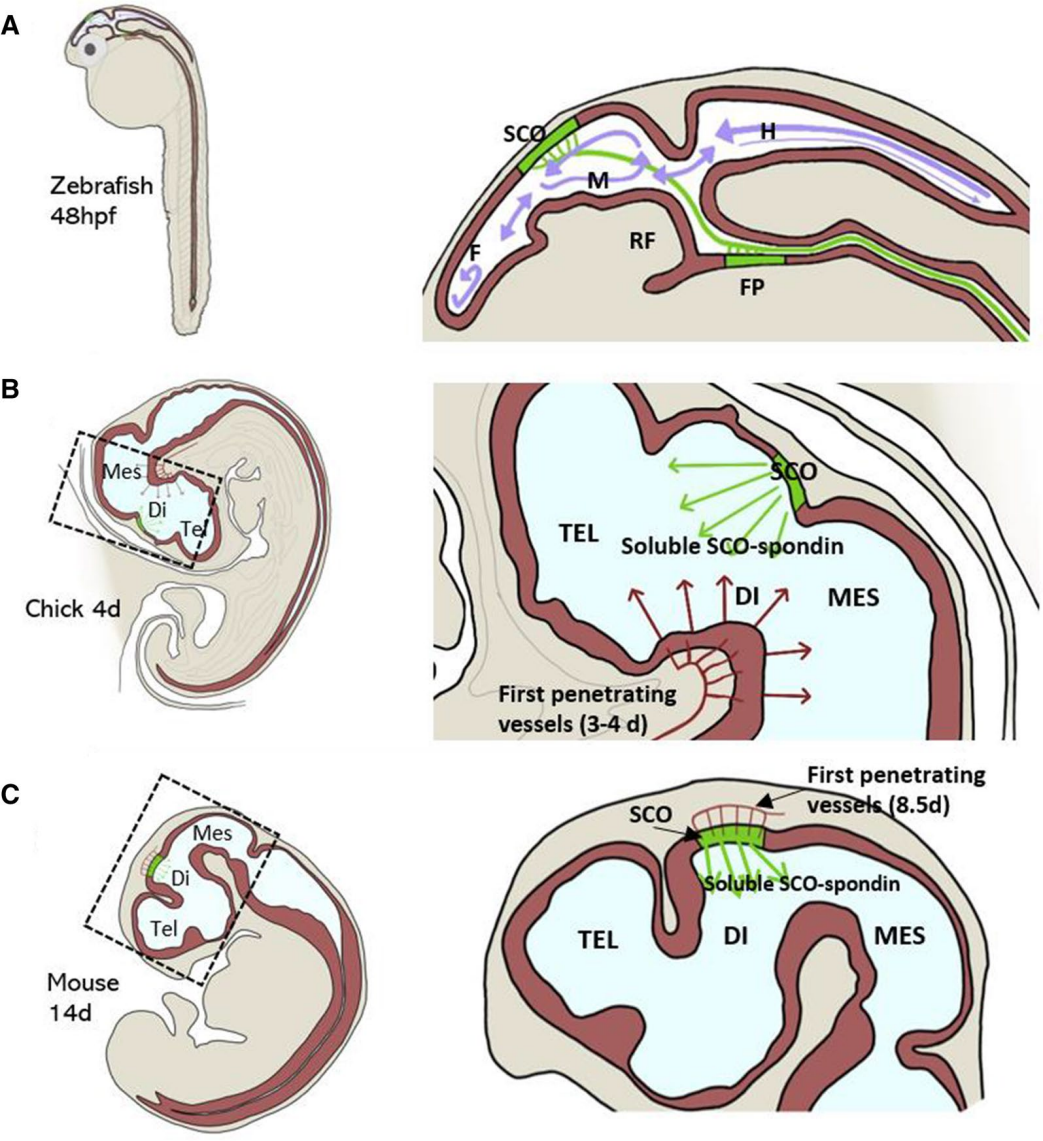
Scheme of the CNS of zebrafish, mouse, and chick embryos, highlighting the localization of SCO. **A** Zebrafish embryos 48 h post-fertilization (hpf). In zebrafish, the RF is formed early in development by SCO-spondin secreted from the SCO and the floor plate. Violet arrows: Direction of CSF flow at this early stage [229]. **B** Chick embryos at 4 days of embryonic development (E4). SCO-spondin is secreted into eCSF from E3.5 and remains soluble until E11, where at least some SCO-spondin aggregates to form the RF. The localization of the first penetrating vessels is shown in red, at the basal region, just in front the SCO [22]. The red arrows represent substances entering to the eCSF through this incipient blood–brain barrier. **C** Mouse embryo at E14. In mouse embryos, the differentiation of the SCO begins at E11, SCO-spondin is secreted into CSF from E14, and the RF forms during the first postnatal week. The first penetrating vessels (in red) enter the mouse brain embryo at the location at which the SCO began differentiating 2 days prior [63]. *Di* Diencephalon, *F* Forebrain, *FP* Floor plate, *H* Hindbrain, *M* Midbrain, *Mes* Mesencephalon, *RF* Reissner fiber, *SCO* Subcommissural organ, *Tel* Telencephalon

**Fig. 4:**
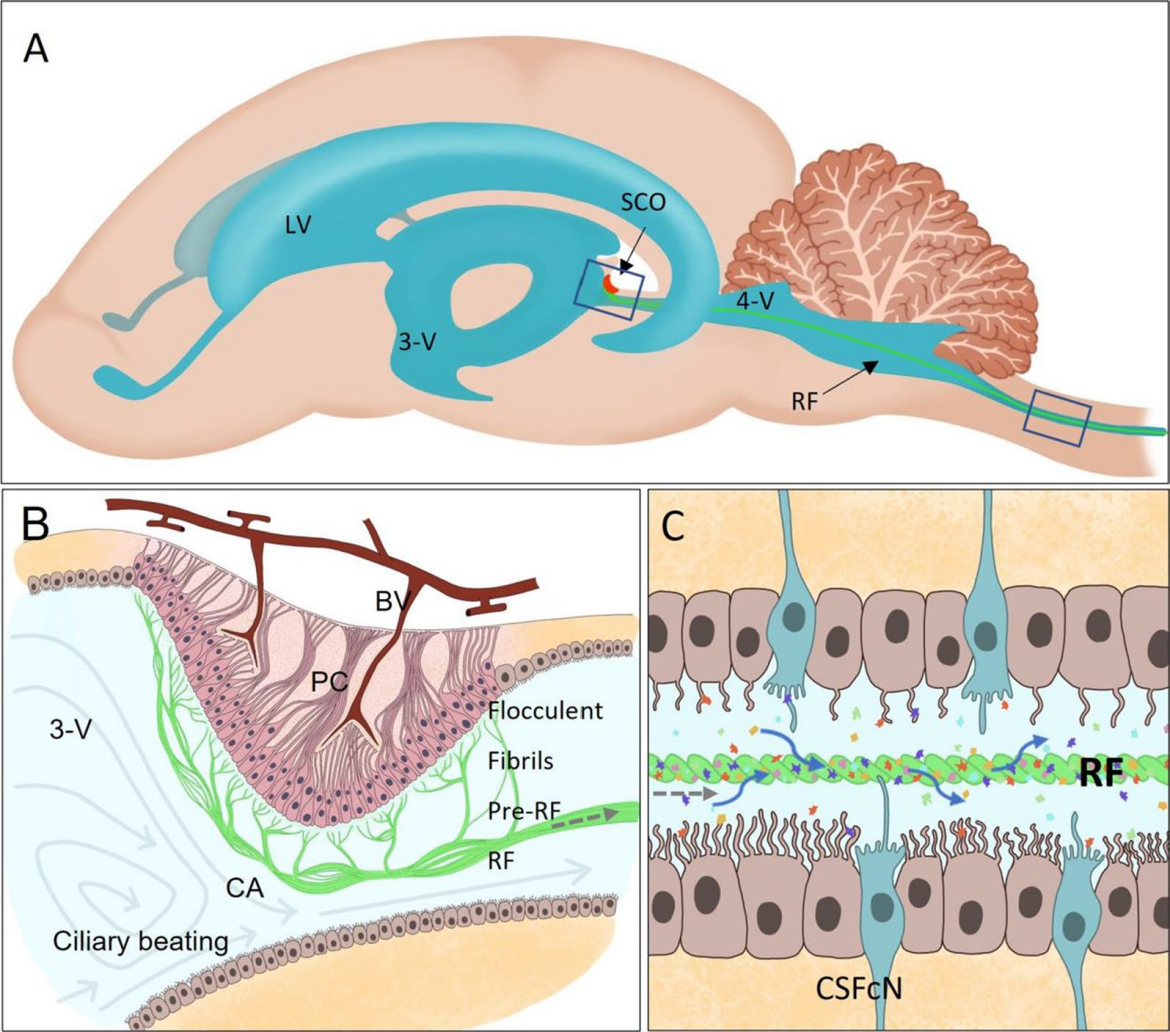
Schematic drawing of rat SCO and RF. **A** Schematic drawing of sagittal section of the adult rat brain showing the cerebral cavities (in blue), highlighting the subcommissural organ (SCO, in red) at the caudal dorsal diencephalon, and Reissner fiber (RF in green) that extends along the cerebral aqueduct (CA), forth ventricle (4-V) and the central canal of the spinal cord. **B** Schematic drawing of a sagittal section of the SCO. The radial cells are arranged in a pseudostratified epithelium composed of a cell body in contact with CSF of the third ventricle (3-V) and a basal process that traverses the posterior commissure (PC) and ends at the external membrane or on blood vessels (BV). At the apical membrane, the SCO-spondin secreted into CSF gradually aggregates to form the RF, first as flocculent material on the cell surface, then as fibrils that aggregate to form the pre-RF, and finally as the RF that reaches the CA. This aggregation requires the ciliary movement of the ependymal cells and CSF turbulence (round arrows) generated at the entrance of the CA. **C** Schematic representation of the RF (in green) inside the central canal of the spinal cord, showing the ciliated ependymal cells, being the motile ventral cilia four times more numerous than the dorsal ones [230] and the cerebrospinal fluid contacting neurons (CSFcN in blue). The RF binds and transports various molecules (see main text for details)

A possible candidate to exert this sensing and modulatory activity is SCO-spondin, a giant CSF glycoprotein, named for the site of secretion at the subcommissural organ (SCO) and its similarity with members of the spondin family, such as TSP, F-spondin, or R-spondin [23] (Fig. 1). SCO-spondin is secreted into CSF since the early stages of development, where it can remain soluble, especially during development [24–26] or aggregate to form a threadlike structure called Reissner fiber (RF) [27, 28], which extends from the diencephalon through the fourth ventricle and runs through the central canal of the entire spinal cord (Figs. 3, 4). The SCO-spondin molecules that form the RF are in continuous movement, as new SCO-spondin molecules are added at its cephalic end and are disaggregated at the caudal end [28, 29]. For instance, a SCO-spondin molecule secreted at the mouse SCO and incorporated into the RF, will reach the tip of the spinal cord 10 days later [28].

Since its description in 1860 [27], the RF has been involved in different biological processes, such as neurogenesis, hydrodynamic balance, CSF flow, morphogenesis, mechanoreception, and CSF transport and detoxification (reviewed in [30–32]), although definitive evidence of some of these roles is lacking. In the same way, the precise composition of RF and the mechanism of SCO-spondin aggregation in order to form the RF are not well understood. However, in the past few years, the study of RF and its mainly component, SCO-spondin, have been addressed by new methodological approaches, revealing some clues about its functional relevance, aggregation process and dynamism [29, 33–37].

In the next sections, we will analyze the principal characteristics of SCO-spondin, including place and regulation of its secretion “SCO-spondin secretion by the floor plate and the SCO” section; its large size and modular structure “SCO-spondin modular structure: a blend of matricellular protein, LDLr family, and vWF polymerization domains” section; its extensive and varied glycosylation “SCO-spondin glycosylation” section; its intrinsic disorder “Intrinsic disorder of SCO-spondin, a structural plasticity relevant to binding” section; possible isoforms and protein cleavages “SCO-spondin, alternative splicing and proteolytic cleavage” section; its multimerization to form the RF “Reissner fiber: composition, formation and movement” section and the function of soluble and aggregated SCO-spondin “Function of the SCO, SCO-spondin, and RF” section.

## SCO-spondin secretion by the floor plate and the SCO

SCO-spondin is highly conserved in all chordates, characterized by the presence of a notochord and a hollow neural tube, structures that arise concomitant to the appearance of RF inside this cavity [38–40]. SCO-spondin is secreted into CSF since the early stages of development, although the place of secretion varies according to the stage of development and the species studied. In cephalochordates and urochordates, SCO-spondin secretion occurs at the infundibular organ, located at the rostral floor plate. This location is maintained in vertebrata embryos, where secretion first occurs at the flexural organ (equivalent to the infundibular organ), and in the floor plate, decreasing at the same time that the SCO begins its secretion, which continues for the entire lifespan of the organism [29, 41–45].

The SCO is an ancient brain gland, located at the midline of the caudal–dorsal diencephalon (Figs. 3, 4A, B). It protrudes toward the third ventricle just at the entrance of the cerebral aqueduct and is one of the first glands to differentiate. The SCO is composed of radial glial cells, with the apical surface in contact with the ventricular CSF, and a long basal process that transverses the posterior commissure and contacts with blood vessels and the lamina terminalis which connects with the subarachnoid space (Fig. 4B). SCO to secrete its products toward the third ventricle from the apical region and toward CSF of the subarachnoid space through the basal processes [28]. Additionally, this location allows the SCO to sense CSF because the narrow entrance of the cerebral aqueduct acts as a funnel and generates turbulence that contributes to mixing of CSF components [46] (Fig. 4B). In this context, the SCO expresses diverse receptors, including FGF receptor 1, 2, and 4 [47] and receptors for melatonin [48], leptin, estrogen [49], aldosterone [50], angiotensin II [51], angiotensin [52], adenosine, imidazoline, glucocorticoids, mineralocorticoids, noradrenaline [53], and prolactin [54]. The physiological relevance of these receptors is not fully understood, but it has been suggested that they control SCO secretion in response of the CSF composition [55]. In this way, amphibian brains treated with aldosterone showed an inhibition of the secretory activity of the SCO [56], and the RF grew faster in the light-adapted than in the darkness-adapted animals [57, 58] probably in response of melatonin. However, the circadian secretion of SCO-spondin in frogs may be also due by the innervation of SCO by neuronal fibers from the pineal gland [59]. It is well-established that the SCO is richly innervated and downregulated by serotonergic fibers, in a lesser extent by GABAergic fibers and poorly innervated by other neuronal systems [60–62]. The SCO location is also relevant during development, when a blood–brain barrier and the choroid plexus are absent, and the first penetrating vessels appear. In the mouse, the first vessels penetrate the location of the SCO at E8.5 [63] (Fig. 3C). In chick, the first penetrating vessels arise in the prosencephalon–mesencephalon ventral region (Fig. 3B), in front of the SCO. These vessels appear around E4 [22], exactly at the moment at which SCO-spondin is detected in the eCSF, allowing interaction between SCO-spondin and the molecules entering the chick eCSF through the ventral region.

Immunohistochemical analysis of SCO in 25 vertebrate species shows a similar organization in all species studied [64], although there is no clear evidence of RF formation in humans, anthropoid apes, or bats. In the case of humans, the SCO is one of the first areas of the brain to differentiate, and the secretion of high molecular weight glycoprotein during fetal and neonatal life is well documented [65, 66]. Since childhood, SCO-specific secretory cells are progressively replaced by a non-secretory ependyma, finding only irregular scattered islets of SCO-cell in a 34-year-old man and a vestigial SCO at older stages [66]. In relation with the secretion of human SCO, it is well documented the absence of RF and a myriad of antibodies made against the bovine RF does not recognize the human SCO. However, the antibody anti P-15 (made against a synthetic 15-mer peptide derived from SCO-spondin sequence) showed intense immunoreactivity in the apical region of the human embryo SCO, where secretion to the eCSF was confirmed by western blot [26], concluding that human SCO-spondin is secreted to the CSF, at least during fetal stages.

## SCO-spondin modular structure: a blend of matricellular protein, LDLr family, and vWF polymerization domains

A general approach to determine the functions of new proteins is to transfer annotations from well-characterized proteins with similar domains, which works even better when there is co-occurrence of several such domains [67, 68]. SCO-spondin is a modular protein, with a molecular weight higher than 500 kDa and composed of several domains with biological relevance (Fig. 1). In chick (UniProt Q2PC93) [69], highly similar to the rest of vertebrates, these domains include one elastin microfibril interface (EMI) domain, three vWF-D domains, one FA5/8C domain, 13 LDL receptor class A (LDLrA) domains, 12 trypsin inhibitor-like (TIL) domains, 27 TSR domains, seven vWF-C domains, three EGF-like domains, and one CTCK domain. The disposition of these domains resembles the summation of CCN matricellular proteins, the ligand-binding region of the LDL receptor family, and the domains responsible for von Willebrand Factor (vWF) aggregation (Fig. 1). Despite the relevance of these domains in other proteins, their function in SCO-spondin remains to be elucidated. In the next sections, we analyze the roles and binding partners described for these domains in other proteins and suggest a possible role for these domains in SCO-spondin, paying special attention to their capacity to bind soluble factors present in CSF, to receptors present in the ependyma, and to domains associated with polymerization, a process necessary to form the RF.

### Matricellular domains in SCO-spondin

Among the SCO-spondin domains, TSR, vWF-C, EGF-like (also present in the LDL receptor family), and CTCK domains are characteristic of matricellular proteins.

#### TSR domain

The TSR domain (IPR000884) is found in several matricellular proteins, such as TSP, R- and F-spondin and all members of the CCN family (Fig. 1). The roles attributed to the TSR domain in TSP-1 include cell attachment, protein–protein interactions, and protein–glycosaminoglycan interactions [70]. Interactors of the TSR domain include transmembrane proteins, such as CD36 and integrins; and extracellular molecules, such as TGF-β, matrix metalloproteinases 2 and 9 (MMP2,9), and FGF2 [71, 72]. For instance, the C-terminus of the TSR domain present in the heparin affin regulatory peptide is responsible for the direct binding to FGF-2, inhibiting its chemotactic role in HUVEC cells [72]. However, under pathological circumstances such as cancer, the TSR domain within TSP-1 mediates tumor growth by interacting with TGF-β and the membrane protein CD36 [73, 74].

There are 27 TSR domains in the vertebrate SCO-spondin protein, suggesting an important role in the biological functions of this protein [40]. In this way, a dodecapeptide derived from the most conserved type 1 TSR sequence promotes neurite outgrowth in neuroblastoma cells by a β1-integrin-dependent mechanism [75] and protects against glutamate neurotoxicity in primary cultures of rat cortical and hippocampal neurons by modulating receptors (integrin B1 and alpha secretase) and intracellular mediators that trigger apoptosis, survival or neurite growth [76]. The same peptide also promotes axonal regeneration/collateral sprouting and subsequent functional recovery in aspiration and contusion models of spinal cord injury in rats [77]. However, this peptide encompassed only a small region of one of the 27 TSR domains of SCO-spondin, suggesting that these domains play more unidentified roles.

As previously stated, the TSR domain in other proteins is a well-established interacting domain for soluble factors, such as FGF-2 and TGF-β, which are also present in CSF (Table 1). FGF-2 is a key eCSF molecule that promotes the proliferation and differentiation of the neuroepithelium [78]. Therefore, the possible binding between FGF-2 and a TSR domain within SCO-spondin could be a regulatory mechanism through which SCO-spondin regulates neurogenic events related to the neuroepithelium. The CD36 receptor, also known as fatty acid translocase, is expressed on the apical region of ependymal cells, which are in contact with CSF [79], and is a high affinity receptor for lipoproteins [80]. The binding of TSR domains from SCO-spondin with this receptor would be important, considering that SCO-spondin also binds LDL from eCSF [81], indicating that this interaction could facilitate binding of LDL to its receptor in ependymal cells.

#### vWF-C domain

The vWF-C (IPR001007) domain, also known as a chordin-like cysteine-rich repeat, is present in several matricellular proteins, including TSP and CCN family members, as well as in other extracellular proteins such as vWF, Chordin family members, and the BMP-binding endothelial regulator.

One of the most reported functions for this domain is the regulation of TGF-β and BMPs [82–84]. The principal effect of this domain in BMP signaling is inhibition and regulation of bioavailability, although in some cases potentiation has been reported [85]. For instance, functional studies of Crossveinless-2 (a member of the chordin family with four vWF-C domains) have shown that BMP binds to the subdomain 1 of vWF-C1 to trigger an anti-BMP effect, whereas direct binding of BMP to chordin via subdomain 2 of vWF-C1 and vWF-C2-4 triggers its pro-BMP effect [84]. TSP-1 also antagonizes BMP2 and BMP4 through its vWF-C domain, probably via the regulation of their bioavailability [85]. CCN2 has been shown to directly bind BMP-4 through its vWF-C domain, impeding its interaction with the receptor, whereas the same domain enhances the binding of TGF-β with its receptor [82]. These BMP-binding proteins might also increase signaling by promoting BMP diffusion and lifespan; in this manner, the same BMP-binding proteins sequester and inhibit BMP signal locally, but increase BMP lifespan and activity range [86], allowing BMPs to travel longer distances and generate gradients with a maintained signal over long periods [87].

Because TGF-β1 and 2 and BMP7 are present in the adult CSF, and BMP activity is detected at the embryonic stage (Table 1), the presence of seven vWF-C domains in SCO-spondin strongly suggests interaction among them. This interaction would be relevant to the concentration, bioavailability, and transport of TGF-β and BMP throughout the entire CNS.

#### Cystine Knot C- terminal domain (CTCK)

The CTCK domain (IPR006207) is a highly conserved three-dimensional folded domain found in several extracellular proteins, including vWF, several mucins, a wide variety of cytokines (e.g. nerve growth factor, TGF-βs, VEGF, BMP antagonists, and slit family proteins), hormones (e.g. luteinizing hormone, chorionic gonadotropin, thyroid-stimulating hormone, and follicle stimulating hormone), and CCN matricellular proteins [88–90]. The consensus sequence of the CTCK motif can be identified by a pattern of six cysteine amino acids within a defined space comprising three intertwined disulfide bridges, two of which form a loop through which the third disulfide bond passes. The rigidity of this domain causes exposure of hydrophobic residues, favoring protein–protein interaction to decrease hydrophobicity [88, 91, 92].

Several interacting partners of the CTCK domain have been identified in other proteins, such as integrins (α6β3, αvβ5, αvβ3, αmβ2, and α5β1), perlecan, vitronectin, decorin, and cell-surface heparan sulfate proteoglycans (HSPGs). In all these interactions, CTCK acts as an important domain that determines how these proteins control cell adhesion processes [93–95]. Additionally, CTCK modulates the Wnt signaling pathway through interactions with LDL receptor related protein 6 (LRP6) [96, 97].

CTCK domain is also involved in the dimerization and polymerization of homo and heterodimers with other proteins containing the same domain [92] and in the formation of long polymers of vWF and mucins [98].

Cumulatively, these antecedents suggest a possible interaction between the CTCK domain within SCO-spondin and factors present in CSF (Table 1) with the same domain, such as nerve growth factor, TGF-βs or HSPGs, as well as its participation in SCO-spondin polymerization to form the RF.

### LDLr family domains in SCO-spondin

All members of the LDLr family share a similar ligand-binding region comprising at least seven LDLrA domains in tandem, followed by two EGF-like domains [99]. The same conformation is found in SCO-spondin, with ten LDLrA domains followed by two EGF-like domains (Fig. 1).

#### LDLrA domain

The LDLrA domain (IPR023415) is distinctive of the LDL receptor family, whose members contain at least seven of these domains in tandem (Figs. 1, 2) crucial for LDL binding activity [100, 101]. The LDL receptor is the prototype of this family, which also includes LDL receptor-related protein 1 and 1b (LRP1-LRP1B), megalina/GP330/LRP2, the VLDL receptor, ApoE receptor-2 (ApoER2), and LRP6. This family of receptors has been linked with several normal and pathological processes of CNS [102].

The binding partners of the LDLrA domain is extensive, and include apolipoproteins (Apo) B, ApoE, reelin, ApoJ (clusterin), TSP, F-spondin, carrier proteins for lipophilic vitamins, proteases/inhibitor complexes, and members of the Wnt family (Table 3) [102, 103].

**Table 3:** Summary of SCO-spondin domains and possible CSF binding partners on the basis of described interactions of these domains in other proteins

Domain	Protein	Ligand (soluble or cell receptor)	References
EMI	Periostin, Emilin-3	Itself, TGFβ1	[118, 119, 210]
	SCO-spondin	Itself	[33]
LDLrA	LDLr	Lipoproteins (Apo B and E)	[102]
	VLDLr	Apo E, Reelin	[102, 103]
	ApoER2	ApoE, Reelin, F-spondin, APP	[102, 211]
	Megalin/LRP2	ApoB, ApoE, carrier proteins for lipophilic vitamins, proteases and inhibitors, Apo J	[102, 212]
	LRP-1	ApoE, APP, protease/inhibitor complex, Thrombospondin-1: β Amyloid peptide, TGFβ, BMP4 MMP2 and 9, Insulin growth factor-1	[102, 213–217]
	LRP6	Wnts	[102, 218]
	SCO-spondin	LDL	[81]
vWFC	Crossveinless	BMP2	[219]
	TSP-1	BMP2,4	[85]
	CCN2	BMP2, TGFβ	[82]
	CCN1	BMPs, Integrin αv β3	[220]
TSR	Thrombospondin-1	MMP-2, CD36, Integrin β1, TGF-β, FGF-2	[74, 221–225]
	CCN Family	Integrin TGFB LRP-1	[17]
	SCO-spondin	Integrin β1	[75]
CTCK	CCN3	Notch1	[226]
	CCN2	HSPG and Integrins α_v_ β_3_	[95]
	CCN1	Wnts and LRP6	[96, 97]
	vWF	Itself	[123]
vWFD	vWF	Itself	[123]
	Gel forming mucins	Itself	[125]
EGF-like	Neuroregulin	Integrins α_v_ β_3_	[227]
	Thrombospondin-1	FGF-1	[110]
	Tenascin	EGF receptor	[111]

LRP1 also binds to the amyloid-β peptide (Aβ), whose accumulation in the brain is a hallmark of Alzheimer’s disease. This receptor is expressed in the brain capillaries and is able to transport Aβ across the blood–brain barrier in a concentration-dependent manner. It has been proposed that the main directionality of the Aβ from the brain to the plasma is owing to the presence of soluble LRP1 in the blood, which acts a “sink,” sequestering 70–90% of the plasmatic Aβ, diminishing its concentration, and favoring the directional transfer from the brain to the blood [104, 105].

The similarity between SCO-spondin and the ligand-binding region of the LDLr family suggests that SCO-spondin binds the same molecules, some of which are present in CSF in normal or pathological conditions, such as Aβ, lipoproteins, clusterin, or reelin (Table 1). In this regard, it has been reported the in vivo interaction between LDL and SCO-spondin in the eCSF [81]. LDL from CSF is critical during early stages of development for the proliferation and differentiation of the neuroepithelium [106]. In vitro, LDL–SCO-spondin interaction diminishes neurodifferentiation induced by LDL in mesencephalic neuroepithelium explants, revealing the modulatory effect of SCO-spondin [81]. Additionally, the participation of lipoprotein particles in the transport of Shh [107, 108] and Wnt5A through the eCSF has been recently reported [109], suggesting the interaction of all these compounds as part of a morphogenic eCSF complex.

In addition to the binding capacities, a hypomorphic missense mutation that disrupts evolutionary conserved cysteine at LDL domain [29] revealed a progressive disassembly of the RF and a possible disruption in the secretion of SCO-spondin from the floor plate, concluding that this domain is also critical for the stability of the RF during zebrafish larval development.

#### EGF-like domain

The EGF-like domain (IPR000742) has been linked to several biological functions and is able to bind different extracellular molecules as well as cellular receptors. In addition to the LDLr family, this domain is also present in some matricellular proteins, such as tenascin and TSP. In TSP, the third EGF-like domain is responsible for FGF-2 binding [110]; in tenascin, EGF-like repeats directly bind to the EGF receptor and activate ERK1/2 signaling [111, 112]. In the LDL receptor family (Figs. 1, 2), this domain, together with the LDLrA domains, forms part of the ligand-binding region [99].

SCO-spondin contains two EGF-like domains following the LDLrA domains, resembling the ligand-binding region of the LDL receptor family. Additionally, this SCO-spondin domain may bind soluble FGF-2 in CSF or the EGF receptor expressed on ependymal cells and in subventricular neurogenic niches [113], where this receptor is involved in the regulation of neural stem cell number and self-renewal [114].

### Polymerization related domains in SCO-spondin

As stated above, SCO-spondin can be found soluble in CSF or as aggregates in the form of RF, an elastic threadlike structure. The process of SCO-spondin polymerization has not been elucidated, but it is interesting that the same domains responsible for the polymerization of vWF (vWF-D, TIL, and CTCK domains), a protein capable of forming ultra-long chains of several hundred of monomers, are also present in SCO-spondin. In addition to these domains shared with the vWF, SCO-spondin also contains one EMI domain, also related with polymerization.

#### EMI domain

The cysteine-rich EMI domain typically contains six or seven cysteine residues, which likely form disulfide bonds. The EMI domain has been identified in few proteins, including elastin microfibril interfacer 1 (EMILIN-1) protein, multimerins, NEU1/NG3, periostin, and TGFβ-inducing protein [115]. In all these proteins as well as in SCO-spondin, the EMI domain is present in a single copy located at the N-terminus [115–117]. The EMI domain is likely responsible for intramolecular disulfide-bridges and intermolecular multimer formation [118, 119].

The role of EMI as a multimerization domain critical for RF assembly is also supported by the analysis of a SCO-spondin zebrafish mutant with five extra amino acids in the single EMI domain, which expresses an abnormal protein that fails to form the RF [33].

#### vWF-D and CTCK domains

vWF-D (IPR001846) is a large domain present in few proteins such as otogelin, zonadhesin, different mucins, vWF, and SCO-spondin, all characterized by the generation of multimers by inter and intrachain disulfide bonds.

**Fig. 5:**
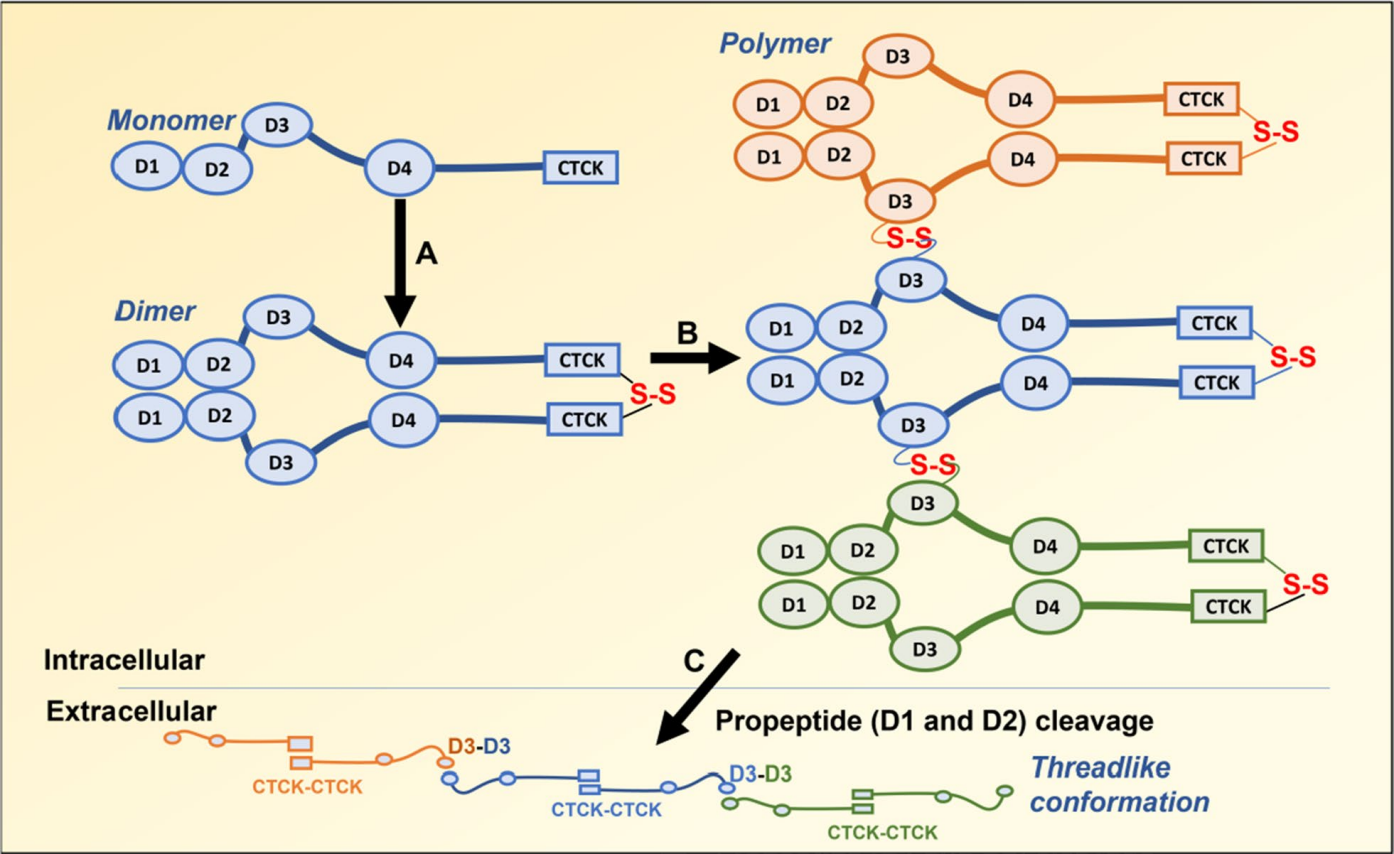
Schematic illustration of von Willebrand factor polymerization, showing the domains responsible of this process. **A** vWF monomers dimerize intracellularly via the interaction of D1 (vWF-D plus TIL domains) and D2 at the N-terminus and the formation of a disulfide bridge between the CTCK domains at the C-terminus of the 2 monomers. **B** The dimers polymerize by the formation of disulfide bridges between D3 regions of adjacent dimers. **C** The region containing D1 and D2 is extracellularly cleaved, and the polymer acquires a threadlike structure

vWF contains four vWF-D domains with a self-organization function (Fig. 5). This protein polymerizes to form long structures critical during the coagulation process, and mutation of the vWF-D domain generates aberrant multimers that lead to a bleeding disorder [120, 121]. In vWF, as well as in mucins, each of the four vWF-D domains is followed by a TIL domain, forming the D1–D4 groups arrangement. Oligomer formation assays revealed that in vWF, D1–D2 are responsible for dimerization at the N-terminus, which is zipped by the interaction among CTCK domains at the C-terminal-end. After dimer formation, the D3 domain forms interchain disulfide bonds with the same domain in an adjacent dimer [98, 122–124]. Once secreted, the propeptide containing the D1-D2 domains is cleaved, causing unzipping of the dimer and leading vWF to acquire a concatenated elongated conformation, which can contain up to 200 monomers, forming a long, flexible, dynamic structure [123]. Therefore, the formation of this long cord of vWF protein relies on three vWF-D domains at its N-terminal end, two of which are removed extracellularly, and a CTCK domain at the C-terminal end. A similar oligomerization process has been described in gel-forming mucins [98, 125]. The same domains are present in SCO-spondin, which contains three vWF-D domains (each followed by a TIL domain) at the N-terminus and a CTCK domain at the C-terminal region (Fig. 1), suggesting that SCO-spondin follows the same strategy of polymerization.

#### Trypsin inhibitor-like cysteine-rich domain (TIL)

The TIL domain (IPR002919) is mainly present in trypsin inhibitor proteins; however, it can also be found in other extracellular proteins, including several mucins [125], the IgGFc-binding protein (nine TIL domains), and the scavenger receptor cysteine-rich protein (six TIL domains) [126]. The principal activity of TIL domain is to inhibit proteinase activity, but it also forms an arrangement with vWF-D domain in some mucins and in vWF [98, 122] where it contributes to the polymerization process.

SCO-spondin contains 16 TIL domains, described previously as SCO-spondin repeats [40], distributed throughout the entire protein and probably contributing to SCO-spondin integrity. Three of these domains are situated after each vWF-D domain, suggesting a role in the SCO-spondin multimerization. In fact, a hypomorphic missense mutation that disrupts a evolutionary conserved cysteine at the second TIL domain [29] generated a progressive disassembly of the RF and a possible disruption in the secretion of SCO-spondin from the floor plate. Similar results were reported after the mutation of other cysteine in the same domain [36], generating abnormal intracellular SCO-spondin immunoreactivity at sites of protein production (even in heterozygotes) and the lack of RF in homozygous embryos, concluding that this mutation may disrupt SCO-spondin secretion and is critical for the stability of the RF during zebrafish larval development [36].

## SCO-spondin glycosylation

In addition to the protein component, SCO-spondin displays a great variety of different N-glycan structures. Electrophoretic analysis of bovine RF treated with endoglycosidase F shows a decrease (between 10 and 25%) in the molecular mass of its four principal immunoreactive compounds [127]. The precise localization of N-glycosylation in this protein is not known, although UNIPROT reveals 44 potential glycosylation sites in chick SCO-spondin.

Recent analysis by multiplexed capillary gel electrophoresis with laser-induced fluorescence detection revealed an extremely complex glycosylation pattern, one of the most intricate found in nature. This pattern ranges from simple neutral biantennary *N*-glycans to highly complex tetra-antennary *N*-glycans containing bisected *N*-acetylglucosamine (GlcNAc), up to three sulfations, and/or several sialic acids of the Neu5Gc or Neu5Ac type [35].

The presence of this abundant and varied negatively charged glycosylation may have important functional consequences. First, it would transform SCO-spondin into a highly polar molecule with relevance in maintaining osmotic pressure. Osmotic pressure is a crucial mechanism to expand the cephalic vesicles during development [128] and may also be important to maintain the opening of narrow cavities, such as the cerebral aqueduct and central canal [129]. Second, its complex glycosylation is similar to that in glycosaminoglycans (GAGs). These molecules consist of disaccharide units frequently modified by sulfation. GAGs interact with various proteins, including soluble proteins (growth factors, morphogens, and chemokines), ECM proteins, bioactive fragments, membrane receptors such as integrins, and lipoproteins [130]. The impacts of GAGs on binding partners are diverse. In some cases, GAGs regulate their activity, acting as a co-factor (like the requirement of heparin for FGF2 function), whereas in other cases, the GAGs may sequester the binding partners, thereby limiting their bioavailability [131]. These antecedents suggest that the glycosylic component of SCO-spondin may be acting in a similar way that GAGs, contributing to the binding and modulation of CSF compounds.

## Intrinsic disorder of SCO-spondin, a structural plasticity relevant to binding

Not long ago, it was believed that all proteins have a well-defined 3D structure related to their unique function. Now, it is known that several proteins lack a stable 3D structure along their entire length or in determinate regions. These proteins have neither regular secondary nor tertiary structures and are dynamic, highly flexible, and disordered under physiological conditions [132]. Intrinsically disordered proteins (IDPs) and intrinsically disordered regions (IDPRs) undergo constant changes by forming hybrids with either ordered or disordered domains, including folded, semi-folded, and unfolded regions, as well as inducible folded regions, depending on the binding partner interaction. Consequently, these proteins exhibit multifunctional behaviors [133, 134]. This structure plasticity also confers the ability to adopt different conformations as they interact with different partners. Contrary to ordered proteins, which fold before becoming functional, IDPs fold at the interaction interface and even after the interaction has completely occurred [133, 135].

IDPRs have a wide range of biological roles. They are important for cell signaling because they can form interaction networks by binding to multiple partners. In this way, several hub proteins are mostly disordered, enabling them to participate in and modulate multiple networks as they bind to multiple ligands [136]. Conversely, IDPRs also act as linkers and spacers, regulating the distance between adjacent domains [137].

Analysis of several extracellular proteins revealed that IDPRs provide structural plasticity necessary for interaction with other molecules. Among the analyzed proteins, the matricellular proteins contain on average a 16.8% of predicted disorder, being the EMI, TSR, vWF-C, and EGF-like domains (all of them present in SCO-spondin) some of the most disorder domains, as they contain high percentages of disorder-promoting residues [138].

The spondin family, including human SCO-spondin, contains several possible disorder-based binding sites with higher degrees of IDPRs in SCO-spondin compared with other spondin family members [139]. SCO-spondin has a range of disorder from 71.2 to 5.4% (evaluated by different IDPRs predictors) with an average of 20–23% (i.e., percentage of residues with disorder scores exceeding the threshold of 0.5). The analysis of IDPR distribution profile in SCO-spondin from different species of vertebrates revealed the presence of IDPRs along its entire length, with a conserved distribution in all species analyzed [139]. These results suggest that intrinsic disorder has a functional importance in SCO-spondin, allowing it to interact with multiple partners or act as a linker/spacer between adjacent domains.

## SCO-spondin, alternative splicing and proteolytic cleavage

One of the characteristics of matricellular proteins is the presence of multiple isoforms generated by alternative splicing and proteolytic cleavage [140–142]. The expression of different isoforms or fragments explains the functional diversity reported for these proteins in several cases. For instance, there are multiple initiation sites in the TN-C mRNA with the potential to generate more than 500 different isoforms through alternative splicing; to date approximately 100 have been reported [143]. Furthermore, TN-C isoforms can be cleaved by members of the MMP family, generating isoforms with specific functions and modulating their interaction with other molecules [144]. Similar results have been reported for TSP-1, which can be cleaved between the vWF-C domain and TSR domain, leading to its release from the extracellular matrix and promoting activation of latent TGF-β [142] or members of the CCN family, cleavage of which regulates the bioavailability and activity of several growth factors [141].

Several imprecise variants of SCO-spondin have been identified in the SCO (place of synthesis), RF (SCO-spondin aggregates) and CSF (SCO-spondin soluble) of vertebrates. Northern blot analysis of SCO using specific probes for SCO-spondin revealed different results, perhaps owing to the probes used, sensitivity of analysis, species, or the developmental stage analyzed. There are two northern blot analysis reports for adult bovine SCO, one describing a unique and strong band larger than 10 kb [145] and the other describing a strong band of 14 kb and minor transcripts of 10 kb, 7 kb and 4.9 kb [146]. In chick embryos, the same analysis revealed a strong band of approximately 15 kb and faint bands of 7, 4, and 2 kb [69]. These results indicate that SCO-spondin may be alternatively spliced, although in a lesser extend than other matricellular proteins considering its enormous size.

At protein level, western blot analyses of protein extracts from the SCO, RF, and CSF revealed a multiplicity of SCO-spondin bands that strongly suggest that SCO-spondin is proteolytically cleaved [24, 25, 147]. In these experiments, the most used antibody is a polyclonal antibody made against the bovine RF. The specificity of this antibody has been confirmed in zebrafish null mutants, in which this antibody does not immunoreacted with any structure, including the floor plate and SCO [36]; and in scospondin-GFP knocking zebrafish in which the label of GFP has a perfect colocalization with this antibody [29]. Additionally, other antibodies against specific SCO-spondin sequences have been used, like anti-p15, made against a synthetic 15-mer peptide derived from bovine SCO-spondin [25].

Western blot analysis using these antibodies showed few bands of high molecular weight (540, 450, and 320 kDa in bovine and 630, 450, 390, and 200 kDa in rat) when protein was extracted from the SCO; however, in the RF and CSF extracts more than 15 bands ranking from 450 to 25 kDa are found in bovine [147] and from 320 to 25 kDa in rat [25]. Moreover, in eCSF of chick embryo, the number and weight of bands immunostained with anti-SCO-spondin depends on embryonic stage [24]. To our knowledge, in humans, there is only one report concerning SCO-spondin in the eCSF, showing seven bands ranging in size from 200 to 25 kDa when the anti P-15 antibody is used [26].

The lack of higher molecular weight SCO-spondin variants in the RF and CSF suggests that SCO-spondin is extracellularly cleaved by unidentified proteases.

Together, these antecedents reveal that SCO-spondin is susceptible to alternative splicing and protein cleavage, suggesting that like other matricellular proteins, these variants have differential roles, such as modifying the bioavailability of binding partners or activation of growth factors.

## Reissner fiber: composition, formation and movement

One of the most fascinating properties of SCO-spondin is its capacity to aggregate and form the RF, a supramolecular structure that traverses caudally from the diencephalon through the cerebral aqueduct, the fourth ventricle, and central canal of the spinal cord (Fig. 4). The RF exists in a state of continuous movement via the addition of new molecules at its cephalic end, which progressively advance until their disaggregation, several days later, at the caudal region of the spinal cord [28, 29] in a movement that resembles a conveyor belt. The daily RF growth rate is different depending on the species studied. For instance, in mouse, the RF grows 10% of its entire length every day; 7% in rat, and 1% in carp; thus, a SCO-spondin molecule secreted at the SCO of these animals will reach the tip of the spinal cord 10 days, 15 days, or 3 months after being secreted respectively [28].

The RF was first described over a century ago [27], and despite the great contributions to our knowledge of this protein made by several research groups, there are several unresolved questions regarding the RF, including the following: What is it composed of? How is it assembled? And What is its function? In the last few years, RF has been studied using new methodological approaches, such as tandem mass spectrophotometry [35] and mutant zebrafish lines [29, 33, 34, 36, 37, 148], providing some insight on these historical questions.

The RF has long been postulated to be composed of SCO-spondin, and it was confirmed by the lack of FR in scospondin mutant zebrafish [33] and by the strong GFP-fluorescence of the RF in scospondin-GFP knocking zebrafish [29]. Additionally, tandem mass spectrophotometry (MS/MS) analysis of the bovine RF revealed that the main constituent of the RF is SCO-spondin; some other proteins did appear in the analysis [35], although there is no certainty whether these proteins are part of the RF or they are bound to the RF. These proteins included clusterin (ApoJ), galectin-1, creatinine kinase B-type, β tubulin 2B chain, α tubulin 1B chain, S100B, S100A1, and calmodulin. Among these proteins, galectin-1 shows immunolocalization within the RF, and its inhibition by injection of antibodies into CSF impeded RF formation, suggesting a role in RF assembly [35]. By contrast, other proteins found in the MS/MS analysis, such as clusterin, appear as possible binding partners, since its interaction with LDLrA domains has been well-established [149].

After its secretion into CSF, SCO-spondin undergoes progressive aggregation, initially as flocculent material deposited on the apical membrane, that undergoes arrangements into fibrils, later as a mesh of fibrils (pre-RF) over the whole SCO surface, and finally as RF, which marks its journey toward the caudal region of the CNS (Fig. 4) [28]. SCO-spondin molecules that conforms the RF are in continuous movement as new molecules are added at its cephalic end. This rostro-caudal movement was initially showed by classical pulse-chase labeling of the RF with radioactive cysteine [150, 151] or radioactive monoamines [152]. Recently, in an elegant study, this process has been showed in vivo by the generation of a scospondin-GFP knocking zebrafish line [29]. This experimental approach confirmed the continuous RF movement, and allowed the visualization of the initial assembly of SCO-spondin to form the RF, revealing that at 20–30 h post-fertilization (hpf) there are a caudal movement of short SCO-spondin fibers from the brain, and SCO-spondin puncta and several boluses of SCO-spondin from the floor plate down the central canal that join with other SCO-spondin-GFP material at the end of the spinal cord, forming a continuous RF between 2 and 3 days post-fertilization.

The progressive aggregation of SCO-spondin has been detailed in bovine RF. Light and electron microscopy revealed a threadlike structure of 50-µm, composed of bundles of thin filaments of approximately 2–5 µm thickness, which in turn are formed by microfilaments of approximately 10 nm thickness that run longitudinally along the fiber [35]. The thickness of the RF varies depending on the species, but the 10-nm microfilaments are maintained throughout the vertebrate phylum, being the structural element of the RF [35].

The mechanisms that cause this high grade of SCO-spondin polymerization are not fully understood, but it seems to have intra and extracellular components. At intracellular level, pulse–chase assays after intraventricular injection 35S-cysteine in adult rats [35] showed that some SCO-spondin molecules rapidly enter the secretory pathway, whereas other SCO-spondin molecules were found some days after the 35S-cysteine pulse in dilated rough endoplasmic reticulum (RER) cisterns. Dilated RER cisterns are common in cells that secrete proteins with several disulfide bridges as well as those that secrete polymeric proteins [153–156]. In these cells, the presence of dilated cisterns is attributable to the initial oligomerization steps, as propeptides impede higher grades of intracellular polymerization. For instance, the initial oligomerization of vWF occurs in the RER and relies on the CTCK domain at the C-terminus and the vWF-D1-TIL (D1) and vWF-D2-TIL (D2) domains at the N-terminus. After the formation of dimers, the third vWF-D3-TIL (D3) domain interacts with the same domain in adjacent dimers, and subsequently, the N-terminus is cleaved, allowing the polymerized vWF to acquire a threadlike structure (Fig. 5) [123]. Similar cleavage of the N-terminal region may occur during SCO-spondin aggregation. This protein is initially synthesized as a precursor protein of 540 kDa, which can be found in the SCO, but not in the RF, where the largest SCO-spondin has molecular weight of 450 kDa [147]. Cleavage at the C-terminal end seems unlikely because the linkage of GFP to this end allowed the visualization of a fluorescent RF [29]. Conversely, the N-terminal region contains the EMI and vWF-D-TIL domains (similar to those in the cleaved region of vWF); moreover, the participation of EMI domain in SCO aggregation has been suggested because the insertion of five amino acids in this domain impaired RF formation [33]. The study on chick SCO-spondin sequence by Procleave, a novel bioinformatic approach [157], revealed hypothetical cleavages sites at positions 914 and 669, with scores of 0.994 and 0.974, respectively, by MMP family. This family of proteases is present in CSF [158] and is involved in the cleavage of other matricellular proteins [159, 160]. This proteolytic activity remains to be confirmed but suggests that SCO-spondin polymerizes in a manner similar to vWF.

At the extracellular level, the progressive polymerization of SCO-spondin is CSF dependent. This requirement seems to have the following three components: first, SCO-spondin may form oligomers at the intracellular level, but the formation of interchain disulfide bridges seems to require other CSF proteins, such as galectin [35]. Second, as stated above, the formation of the RF requires partial proteolysis of the secreted oligomers. Third, the contribution of CSF flow to RF assembly has been confirmed in mutated zebrafish embryos with deficits in cilium motility, in which the RF cannot assemble despite correct SCO-spondin secretion [29, 33]. At this respect, is important to highlight that SCO is located at the entrance of the narrow cerebral aqueduct, characterized by the presence of turbulences [46] and that extensional flow can catalyze the partial/full unfolding of proteins, exposing previously sequestered protein sequences whose aggregation propensity determines the probability and extent of aggregation [161].

Once formed, RF exists in a state of continuous movement via the addition of new molecules at its cephalic end, which progressively advance until their disaggregation, several days later, at the caudal region of the spinal cord [28]. In this manner, the daily RF growth rate is different depending on the species studied. For instance, in mouse, the RF grows 10% of its entire length every day; 7% in rat, and 1% in carp; thus, a SCO-spondin molecule secreted at the SCO of these animals will reach the tip of the spinal cord 10 days, 15 days, or 3 months after being secreted respectively [28].

In summary, the formation of the RF is a complex process, which may require initial intracellular SCO-spondin oligomerization, extracellular SCO-spondin cleavage, interaction with galectin, and polymerization in growing structures (microfilaments, filaments, bundles, and FR) dependent on CSF flow.

## Function of the SCO, SCO-spondin, and RF

The function of the SCO and its secreted product, SCO-spondin, has remained elusive for more than a century. Multiple possible functions have been attributed, with the most relevant being different aspects related with morphogenesis, CSF cleaning and transport, maintenance of CSF flow, and prevention of hydrocephalus [28, 30, 31, 38]. These functions are in accordance with the proposed function of SCO-spondin as a matricellular protein, which depending on the isoform, binding partners, and physiological context, can have multiple functions. Recently, with the advancement of microscopy and implementation of new molecular biology techniques, some of these historically suggested functions have gained support.

### Morphogenesis: neurogenesis, axon guidance, and straight body axis

SCO-spondin is expressed early during development, but at a variable stage depending on the species; for instance, 17 h post-fertilization (hpf) in zebrafish, 3.5 days in chick, and 14 days in rat [29, 162, 163]. In the same manner, its aggregation to form the RF also varies, with some species exhibiting concomitant secretion and aggregation, whereas in others, the formation of RF occurs days or even weeks after the first secretion of SCO-spondin; for instance, 20 hpf in zebrafish, 11 days in chick, and first postnatal week in rat [29, 162, 163]. It is interesting to highlight that in aquatic animals that need to rapidly acquire the correct axis and swimming competence, RF formation begins early in development, whereas in contrast, in mammals or birds, the formation of the RF is delayed, and SCO-spondin remains soluble in eCSF meantime (Fig. 3).

**Fig. 6:**
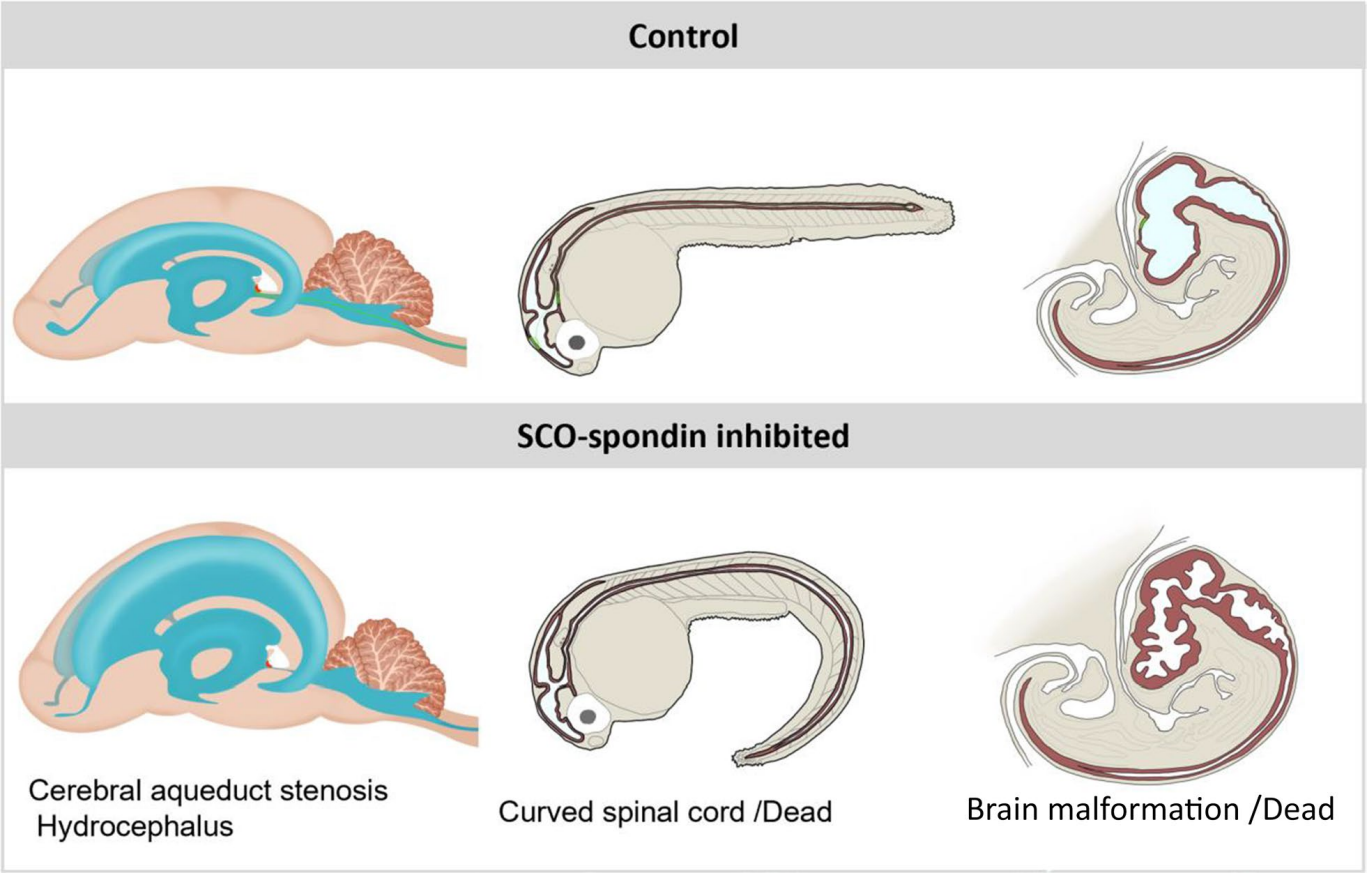
Schematic illustration of the phenotype of SCO-spondin-deficient animals. Hydrocephaly is described in mammals, the curved spinal cord described in zebrafish embryos, and the severe brain malformations described in chick embryos (See main text for details)

The inhibition of SCO-spondin using diverse approaches showed the relevance of this protein at various embryonic stages and different animal models (Fig. 6). In this manner, chick embryos electroporated with SCO-spondin RNAi presented high neuroepithelium proliferation at the expense of inhibition of the neurodifferentiation process. These embryos showed serious malformations throughout the entire brain and died some days after electroporation [24]. In chick embryo, SCO-spondin remains exclusively soluble from 3.5 day until day 11, when it begins to aggregate to form the RF [162]. Immunohistochemical analysis revealed that during this period, SCO-spondin binds to the apical membrane of neuroepithelial cells [24]. The neurogenic function of SCO-spondin is also supported by in vitro experiments in which solubilized RF or peptides derived from SCO-spondin promoted the survival and differentiation of neuronal cells [32, 164–166]. Similarly, mesencephalic explants maintained in eCSF exhibited a drastic decrease in neurodifferentiation after the addition of anti-SCO-spondin antibodies into the culture medium [24] (Table 2). The neurodifferentiation promoted by SCO-spondin is mediated, at least in part, by its capacity to bind and regulate other CSF factors, such as LDL, supporting again its matricellular function [81].

The relationship between abnormal RF and curved body axis has been historically reported in fishes and lizards [167, 168] although until recently the cause-effect relationship was not clear. In zebrafish, the secretion of SCO-spondin begins at 17 hpf and is almost simultaneous with its aggregation to form the RF. In an elegant study, Cantaut-Belarif et al. [33] generated the first SCO-spondin (sspo) mutant using CRISPR/Cas-9-mediated genome editing. The homozygous mutant embryos failed to assemble the RF, developed curled-down axes, and finally died approximately 10 days post-fertilization. This phenotype resembles the curly tail phenotype described for cilia motility mutants, although sspo mutants display normal cilia motility and eCSF flow. By contrast, mutant animals with altered cilia motility and normal SCO-spondin fails to form the RF, suggesting that RF formation requires CSF flow generated by motile cilia. The curly phenotype of embryonic SCO-spondin mutants and the embryonic lethality of this mutation were confirmed by Rose et al. [36] (Fig. 6). In these null SCO-spondin mutants, the removal of the chorion at 24hpf produced embryos with less severely curved axis and approximately 30% of these dechorionated embryos survive and matures into adult fish with strong curvatures of the spine [37]. In the same way, the relevance of the RF in the maintenance of straight body axis was revealed by the generation of two hypomorphic zebrafish mutants, in which an intact RF develops up to 5 days, but it begins to disassemble after a week, coinciding with the appearance of axial curvature in these animals [29].

Transcriptomic analysis of sspo zebrafish mutants [36, 148] revealed high downregulation of urotensin neuropeptide 2 (Urp2). This peptide is secreted in the spinal cord by ventral cerebrospinal fluid-contacting neurons (CSFcNs), a group of mechanosensory neurons that extend motile cilia and microvilli toward the central canal that will eventually contact the RF, detecting the spinal curvature in a directional manner (Fig. 4C) [34]. The secretion of Urp2 by CSFcNs [148] is stimulated by epinephrine and norepinephrine, molecules that are bound on RF surface [152]. The relevance of Urp2 lies in the fact that its expression can restore the axis defects shown in sspo mutants. In the same way, the exposure of sspo mutants to epinephrine and norepinephrine increased Urp2 expression, thereby restoring straight body axis [37, 148]. These results demonstrate the relevance of the RF in the transport of molecules throughout the entire nervous system.

SCO-spondin also participates in axon guidance in the posterior commissure (PC). This commissure is located between the basal processes of SCO cells, and immunohistochemical analysis suggested that SCO-spondin is also secreted by these processes toward the extracellular space, aiding in the guidance and fasciculation of the PC axons [24, 169–171].

In summary, SCO-spondin is crucial in morphogenesis, and its mutation causes severe malformations and embryonic lethality. These malformations are found in the cephalic cavity of embryos in which SCO-spondin remains soluble (e.g. in chick) and in the spinal cord of embryos where the SCO-spondin rapidly aggregates to form the RF (e.g. in zebrafish). These malformations can be explained, at least partially, by the ability of SCO-spondin to bind LDL [81], epinephrine, and norepinephrine [148, 152].

### CSF flow and hydrocephaly

CSF flow depends on multiple factors, such as CSF production by the choroid plexus, ciliary beating of ependymal cells, heartbeat, and pulsatile and local exchange among interstitial fluid, blood, and CSF [4, 172]. In addition to these factors, CSF flow, SCO development, and RF formation seem to have a mutual interdependence. Animals with ciliopathy, in which CSF flows abnormally, fail to form the RF and develop severe malformations [33] or hydrocephaly [173]. The requirement of correct CSF flow for the formation of the RF is not well understood, but it has been suggested that the turbulence of the flow plays an important role, resembling the requirement of blood flow for vWF polymerization. Conversely, at least in mammals, there is a correlation between SCO-spondin and the cerebral aqueduct opening; thus, individuals develop hydrocephaly when the SCO development or the RF formation is impaired [129]. In this regard, immune-mediated blockage of the SCO and RF in rats by the maternal transfer of anti-SCO-spondin antibodies leads to stenosis in the cerebral aqueduct and appearance of hydrocephalus (Fig. 6) [174]. Similar findings have been reported in the human fetal hydrocephalic brain, which also exhibits SCO alterations [175], abnormalities in SCO-spondin secreted into CSF, and occlusion of the cerebral aqueduct [26]. A proposed explanation of this pathology is that the highly charged negative glycosylation of SCO-spondin may generate an electrostatic repulsion on the ependymal walls [26, 129], impeding the collapse of this narrow aqueduct. On the other hand, when RF formation is altered in adult rats by injecting antibodies against RF into the third ventricle, the main CSF flow in the central canal of the spinal cord decreases, as well as the uptake of CSF soluble molecules by ependymal cells [176].

### Matricellular function, a conveyor belt inside the CNS

As stated in the introduction, CSF composition must be finely tuned, establishing a stable internal milieu for the brain, but it is a dynamic fluid, which transports nutrients, neuroactive substances, and waste substances for clearance across the entire CNS. This apparent paradox can be explained by mechanisms capable of binding and releasing CSF factors depending on physiological context. SCO-spondin and the RF appear to be optimally suited for this job owing to the multiple potential binding sites for CSF factors (Table 3, and references therein) and the movement of the RF that allows bound molecules to move toward the caudal region.

To date, there is evidence regarding the binding of monoamines [152] and LDL [81] to SCO-spondin. These bindings seem to be reversible and concentration-dependent, presenting the possibility that SCO-spondin acts as a concentration regulator of these molecules, and in doing so, participates in the homeostasis of CSF. In this regard, it was possible to detect tritiated serotonin and norepinephrine attached to the RF after their injection into the rat lateral ventricle. Initially, these amines were detected in the cephalic RF region, but 1 week after the injection, these amines were found on the surface of the most caudal region of the RF, reaching the tip of the spinal cord. The intensity of the autoradiographic stain revealed that as the RF moved along the central canal, these amines progressively detached [152].

The function of SCO-spondin as matricellular protein is also supported by transcriptome analysis of sspo mutant zebrafish. It is well-established that the loss of a gene in a mutant animal is frequently compensated for by another with overlapping functions, triggering a transcriptional adaptive response [177]. In this manner, transcriptome analysis of mutant sspo zebrafish embryos revealed that genes involved in transport or neuromodulation, such as apolipoprotein a4, the ADAM metallopeptidase with a TSR motif, suppressor of cytokine signaling 3, cerebellin, a Wnt signaling pathway inhibitor, and solute carrier family 13, are among the most overexpressed genes [36, 148], supporting the role of SCO-spondin in the transport and modulation of diverse CSF molecules, including waste substances.

The relevance of SCO-spondin in CSF homeostasis is also suggested in scoliotic scospondin^dmh4/+^ mutant zebrafish [36]. These animals present a severe disruption in the SCO-spondin localization at juvenile stages, with ectopic SCO-spondin accumulation in the brain cavities and a lack of RF. The RNA seq analysis of brains isolated from these animals revealed an upregulation in genes that govern inflammatory and oxidative stress responses. Localization of proinflammatory cytokines in these animals revealed an increment in the telencephalon, confirming the neuroinflammatory response [36]. Having into account that the telencephalon is not in contact with RF, this result suggests that the neuroinflammatory response are due to defects in the activity of SCO-spondin soluble or alterations in CSF homeostasis.

The detoxification role of SCO-spondin has also been suggested in animal models of copper [178], aluminum [179], and lead [180] intoxication. In these animals, acute or chronic metal exposure led to a reduction in the secretion of RF material, suggesting that this decrease, at least in part, causes toxicity. In these cases, treatment with curcumin leads to restoration of RF secretion parallel with an improvement in the toxicity symptoms.

## Conclusions and perspectives

The background set out above suggests that SCO-spondin is a giant matricellular protein capable of maintaining homeostasis in CSF by modulating and trapping several binding factors and permitting the physical dynamism necessary for transporting and releasing the bound molecules in different spatial, temporal, and biological contexts. The binding of SCO-spondin to some relevant molecules has been already reported; however, considering that other smaller matricellular proteins bind more than 80 different molecules, it seems that only the tip of the iceberg of binding partners has been discovered. In this review, we suggest more than 30 possible binding partners (Table 3), including Aβ and several growth factors, interactions that deserve to be studied.

In relation to the SCO-spondin in humans the information is contradictory. Human SCO is well developed during fetal and neonatal stages, but it progressively regresses at posterior stages [66]. The secretion of SCO-spondin has been reported at fetal stages [26], but there is no information about posterior stages and the RF is not formed at any stage. UniProtKB database reveals the occurrence of human SCO-spondin at transcriptomic level (A2VEC9) although the Human Genome Organization Gene Nomenclature Committee classifies human SCO-spondin as pseudogen (HGNC: 21998). The relevance of clarifying this aspect lies in the fact that anormal human SCO-spondin has been linked to several pathologies, including hydrocephaly [26, 175], Parkinson’s disease [181], phenylketonuria [182], cancer [183], congenital midline cervical cleft [184], and schizophrenia [185]. In relation to schizophrenia, is interesting to highlight that this disease is related with cerebral aqueduct stenosis [186] and hydrocephaly [187], two pathologies also associated with SCO-spondin anomalies [26, 175]. It has also been proposed that the progressive atrophy of human SCO with the subsequent lack of SCO-spondin may be contributing to the failure of human adult neurons to repair in CNS injuries or diseases [76]. In this way, a peptide derived from the first TSR domain of SCO-spondin protects neurons from glutamate-induced excitotoxicity [76] and restore learning and memory in a mouse model of Alzheimer’s disease [188].

Full understanding of SCO-spondin properties, including its structural conformation, regulation, and behavior in different contexts, will lead to a better comprehension of CSF physiology and open possibilities to new therapeutic tools for treating pathologies. In fact, the binding capacities of matricellular proteins and GAGs have already been exploited in drug delivery for the treatment of different diseases [131, 189].

In summary, SCO-spondin is an incredible protein, highly conserved, highly glycosylated, highly disordered, with several isoforms and enormous size. The study of this protein is complex but extremely relevant. Thus, with this review, we aim to motivate new researchers in the field to better understand this ancient and versatile protein.

## Data Availability

Not applicable.

## Abbreviations

3-V: Third ventricle
4-V: Fourth ventricle
ApoB: Apolipoprotein B
ApoE: Apolipoprotein E
ApoER2: ApoE receptor 2
ApoJ: Apolipoprotein J (clusterin)
Aβ: Amyloid-β peptide
BMP: Bone morphogenetic protein
BV: Blood vessels
CA: Cerebral aqueduct
CCN: Acronym for **C**onnective tissue growth factor (CTGF), **C**ysteine rich protein (Cyr61), and **N**ephroblastoma overexpressed gene (nov)
CNS: Central nervous system
CSF: Cerebrospinal fluid
CSFcNs: Cerebrospinal fluid-contacting neurons
CTCK: Carboxyl-terminal cystine knot domain
CTGF: Connective tissue growth factor
Cyr61: Cysteine rich protein
ECM: Extracellular matrix
eCSF: Embryonic CSF
EGF: Epidermal growth factor
EMI: Elastin microfibril interface domain
EMILIN-1: Elastin microfibril interfacer 1
FGF: Fibroblastic growth factor
GAGs: Glycosaminoglycans
GFP: Green fluorescent protein
GlcNAc: *N*-Acetylglucosamine
Hpf: Hours post-fertilization
HSPG: Heparan sulfate proteoglycan
IDPRs: Intrinsically disordered regions
IDPs: Intrinsically disordered proteins
IGFBP: Insulin-like growth factor-binding protein
LDLr: Low density lipoprotein receptor
LDLrA: LDL receptor class A domain
LRP1: LDL receptor related protein 1
LRP1B: LDL receptor related protein 1B
LRP6: LDL receptor related protein 6
LV: Lateral Ventricle
MMP: Matrix metalloproteinase
MS/MS: Tandem mass spectrometry
NEU1: neuraminidase 1
Neu5Ac: *N*-Acetylneuraminic acid
Neu5Gc: *N*-Glycolylneuraminic acid
Nov: Nephroblastoma overexpressed gene
PC: Posterior commissure
RBP: Retinol binding protein
RER: Endoplasmic reticulum
RF: Reissner fiber
RNAi: RNA interference
SCO: Subcommissural organ
SSPO: SCO-spondin gene
TGF-β: Transforming growth factor- β
TIL: Trypsin inhibitor-like
TNC: Tenascin
TSP: Thrombospondin
TSR: Thrombospondin type I repeat
Urp2: Urotensin neuropeptide 2
VEGF: Vascular endothelial growth factor
vWF: Von Willebrand factor
vWF-A, C and D: VWF type-A, C and D domain respectively
